# Proteomic and Phosphoproteomic Analyses Reveal a Complex Network Regulating Pollen Abortion and Potential Candidate Proteins in TCMS Wheat

**DOI:** 10.3390/ijms23126428

**Published:** 2022-06-08

**Authors:** Liting Ma, Yuran Hao, Xiaorong Liu, Leilei Shao, Hairong Wang, Hao Zhou, Dazhong Zhang, Ting Zhu, Qin Ding, Lingjian Ma

**Affiliations:** 1 College of Agronomy, Northwest A&F University, Yangling 712100, China; 2018060009@nwafu.edu.cn (L.M.); yrhao@nwafu.edu.cn (Y.H.); lxr19980722@nwafu.edu.cn (X.L.); 2021060015@nwafu.edu.cn (L.S.); 2019055037@nwafu.edu.cn (H.W.); 2019050046@nwafu.edu.cn (H.Z.); zdz1727697945@163.com (D.Z.); zhuting@nwafu.edu.cn (T.Z.); 2 College of Horticulture, Northwest A&F University, Yangling 712100, China; dingqin@nwafu.edu.cn

**Keywords:** anther, pollen, proteomics, phosphoproteomics, male sterility, wheat

## Abstract

Thermosensitive sterile lines are natural materials for exploring the effects of anther development on male fertility. To study the possible molecular mechanisms regulating protein activity during the induction of male sterility, proteomic and phosphoproteomic analyses with tandem mass tags (TMTs) were used to study the binucleate anther of the thermosensitive sterile wheat line YS3038. A total of 9072 proteins, including 5019 phosphoproteins, were identified. Enrichment analyses of differentially abundant proteins (DAPs) and phosphoproteins (DAPPs) in metabolic pathways showed that both were mainly related to energy metabolism. Soluble sugar and ATP content were significantly decreased, free fatty acid content was significantly increased, and ROS was abnormally accumulated in male sterile YS3038-A. In addition, 233 kinase–substrate pairs involved in potential phosphorylation control networks were predicted to regulate fertility. Candidate proteins were identified, and a quantitative real-time polymerase chain reaction (qRT-PCR) analysis was used to validate the TMT results. *TaPDCD5* is likely to be involved in fertility conversion of YS3038 by barley stripe mosaic virus-induced gene silencing (BSMV-VIGS). Our data provide new insights into the mechanism of TCMS, which has value for identifying potential candidate proteins associated with the formation or abortion of pollen and promotion of wheat heterosis utilization.

## 1. Introduction

Heterosis is an important mechanism used to increase crop yields. Owing to a lack of sterility genes, the scarcity of available sterile lines and difficulties in their extensive utilization, and the restricted scale and application of hybrid wheat production, the global planting area of hybrid wheat is less than 1% of the total planting area of all wheat [1]. Increasing the production of wheat still depends on traditional breeding methods, but the utilization of heterosis has become a critical potential means of improving wheat yield, resistance, and adaptability [2]. The heterotic advantage of some crops, including maize CMS-T and Chinese hybrid rice, relies mainly on cytoplasmic male sterility (CMS) [3]. CMS plays a vital role in the production of hybrid seeds and facilitates environmental protection without requiring emasculation [4]. The development of wheat CMS lines revealed new potential for the utilization of wheat heterosis. At present, thermo/photoperiod-sensitive genic systems and CMS are being studied to explore their application potential in wheat [5,6]. Thermo-sensitive CMS (TCMS) wheat can be used to maintain and propagate male sterile lines via self-pollination, and it can produce a large number of seeds in some areas where the average daily temperature can consistently reach the fertile environmental temperature. Additionally, TCMS lines can be used as seed parents when male sterility is induced in other areas, so it has broad application prospects [7]. TCMS not only provides important breeding conditions for utilizing hybrid advantage in crops, but also provides researchers with natural materials for manipulating cytonuclear interactions and exploring male sterility.

The development of anthers is a key process that directly determines the male fertility of plants, and understanding the molecular mechanisms of anther development is important for the improved use and production of new CMS lines. Wheat male sterility is a complex developmental process that includes the differentiation of tissues and the coordinated expression of genes. We performed transcriptome sequencing of tissue from YS3038 (a TCMS wheat line). As it is known that the relationship between the levels of mRNAs and the abundance of the protein products is nonlinear [8,9], estimates of transcription levels are insufficient for explain the final gene expression results, and most of the gene’s function is ultimately realized in the form of protein [9]. Therefore, further study of the molecular mechanism of male sterility in wheat is merited. Contemporary proteomic techniques provide powerful high-throughput methods for identifying molecular mechanisms related to CMS. Moreover, newly synthesized proteins that have undergone post-translational modification (PTM) increase the diversity of protein functions, meaning that some proteins without biological function could become functional proteins after PTM [10]. Protein phosphorylation and dephosphorylation are highly controlled biochemical processes that are closely related to plant growth, development, and resistance to various stresses. Phosphorylation status modulates protein activity, influencing the structure of a protein, controlling its subcellular distribution, and regulating its interactions with other proteins [10]. Phosphorylation is a dynamic process, and the quantitation of these phosphorylation events is crucial [11]. The phosphorylation of some meiotic-related proteins, secretory proteins, kinases, and epigenetic-related proteins has been observed and is considered to be an important factor in anther development in rice [12]. A proteomic analysis of two CMS lines of rice revealed that their pollen was sterile due to a lack of NAD+-dependent malic enzymes, while the up-regulation of acetyl-CoA synthetase and isoamylase may be strongly related to CMS and amylose content [13]. Studies on anther development in Arabidopsis have shown that the phosphorylation of mitogen-activated protein kinases, casein kinase II, and 14-3-3 proteins is a key regulatory mechanism involved in anther development [14]. During pollen development of CMS kenaf, a total of 3045 phosphosites were identified, with roles in carbohydrate and energy metabolism, signal transduction, and cell cycle control, which might in turn play key roles in pollen development [15]. Cysteine protease was observed to promote cell death in the anthers of male sterile transgenic tobacco plants, and the carboxylesterase17 and patatin-like protein-2 in anthers stimulated cell death, likely through the coordination of gene silencing mechanisms and lipid metabolism [16]. These studies elucidate the mechanisms of anther development and improve our understanding of the molecular mechanisms of male sterility.

Few studies have been published on the total protein expression and phosphorylation level of wheat anthers [11]. The total protein expression level and PTM of wheat temperature-sensitive male sterile anthers remain essentially unknown. Therefore, using proteomic and phosphoproteomic tools to discover the mechanism of anther and pollen development will enable researchers and breeders to better utilize and produce novel CMS lines. YS3038 is a temperature-sensitive sterile line of wheat created by our research group. Based on previous studies, we considered the binucleate stage to be a critical period for male sterility in YS3038 [17]. The objective of the present study was to identify candidate proteins that may contribute to wheat sterility by comparing fertile and sterile anthers, obtaining key differentially expressed proteins and phosphoproteins, and identifying patterns of protein regulation among significantly enriched pathways involving male sterility. These results lay the foundation for exploring the genetic and molecular mechanisms of male infertility in wheat.

## 2. Results

### 2.1. Phenotypic Characterization and Cytological Observations

**Figure 1:**
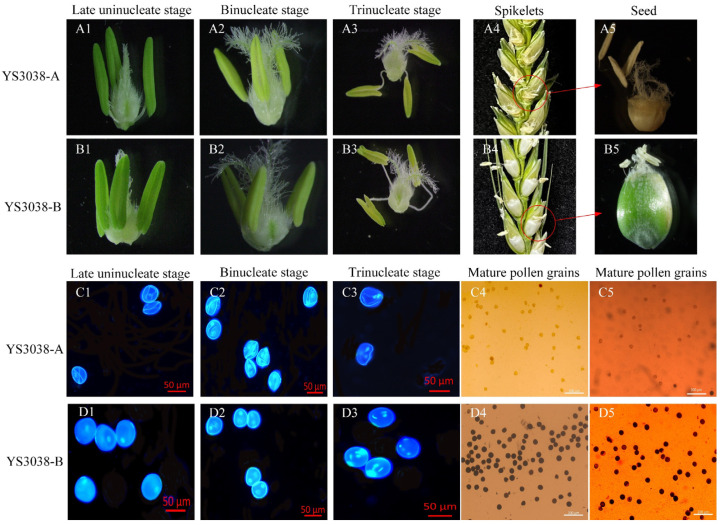
Phenotypic and cytological observations. (**A1**–**A5**,**B1**–**B5**) Anther structure, the morphology of the seed status on spikelets and the seed status in YS3038. (**C1**–**C5**,**D1**–**D5**) Microspores DAPI staining of microspores, I_2_-KI and peroxidase staining of mature pollen grains. (**A1**,**B1**,**C1**,**D1**), Late uninucleate stages. (**A2**,**B2**,**C2**,**D2**), Binucleate stages. (**A3**,**B3**,**C3**,**D3**), Trinucleate stage. Scale bars = 50 µm (**C1**–**C3**,**D1**–**D3**), 200 µm (**C4**,**C5**,**D4**,**D5**).

Anthers and microspores could maintain normal development from the meiotic stage to early uninucleate stage in YS3038-A (Supplementary Materials Figure S1). After the binucleate stage (Bns), anthers of YS3038-A were thinner, and anthers did not dehisce at the trinucleate stage. The pollen of YS3038-A became completely sterile after panicle differentiation. Thus, the microspore development of YS3038-A was defective. YS3038-A pollen was found to be almost entirely aborted by I_2_-KI and peroxidase staining (Figure 1).

### 2.2. Proteomic and Phosphoproteomic Identification

**Figure 2:**
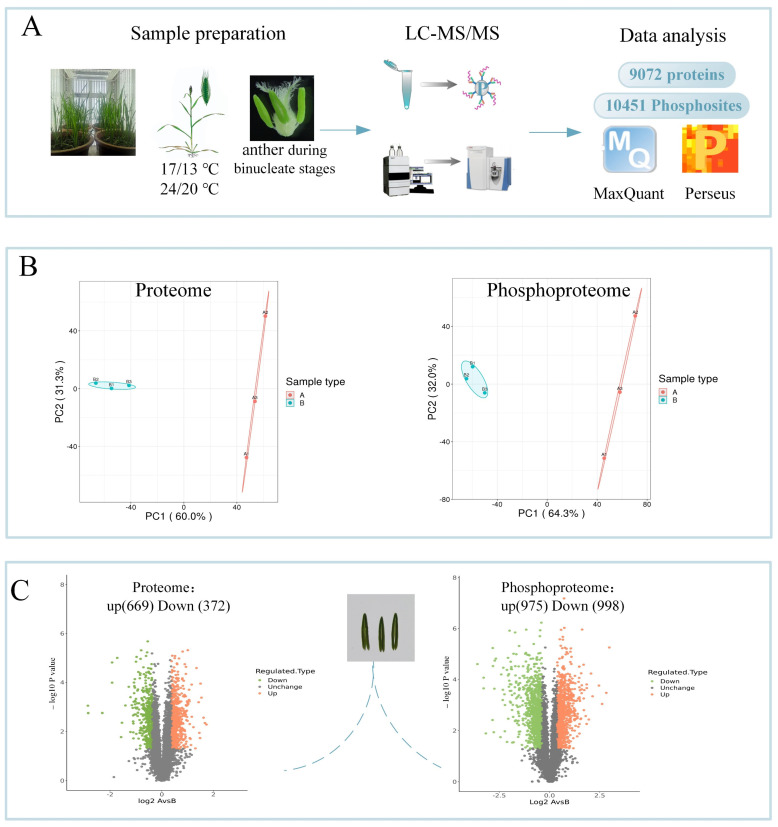
Experimental strategy. (**A**) Sample preparation and workflow of the integrated proteomic and phosphoproteomic analyses. (**B**) Scatter plot of quantitative principal component analysis between repeated samples in the proteome and phosphoproteome data. (**C**) Up- and down-regulated proteins and phosphosites that were statistically significant are represented in the scatter plots. Each point represents a protein or a phosphosite.

The proteomic and phosphoproteomic identification assays were highly accurate, and the results of tests of repeatability are summarized in Figure 2 and Supplementary Materials Figure S2. A total of 9072 proteins, 669 up-regulated proteins and 372 down-regulated proteins (Supplementary Materials Table S1), were identified in the proteome. Additionally, 5019 unique phosphoproteins with 10451 phosphosites (Supplementary Materials Table S2), 975 up-regulated phosphosites and 998 down-regulated phosphosites, were observed in the phosphoproteome (Supplementary Materials Table S3). Further analysis showed that 2869 phosphoproteins possessed only one phosphosite. Two phosphoproteins (W5FM32 and A0A3B6HPU7) with unclear functions possessed 27 and 32 phosphosites, respectively. Compared with previous studies on cereal crops, more proteins and phosphosites were detected in the present experiment [8,11,18,19,20] (Supplementary Materials Figure S3).

Most phosphorylation events in YS3038 anthers occurred at serine residues (9493, 90.8%) followed by threonine residues (918, 8.8%), while phosphate tyrosine residues accounted for 0.4% of the identified phosphosites (Supplementary Materials Figure S4). By comparing the results of the proteomic and phosphoproteomic datasets, regardless of protein abundance, phosphosites were detected without bias. The identified proteins were divided into three subgroups; besides structural proteins, most functional proteins, especially regulatory proteins, were not abundant in the anthers, although they play a crucial role in anther development. Only 43 phosphoproteins were annotated as regulatory, meaning that the functional effects of the vast majority of phosphorylation events quantified here or in upstream kinases have not yet been studied. At present, the ProteomeXchange Consortium has no wheat datasets related to anthers, and our research appropriately fills this gap. The data obtained in this study were deposited in the ProteomeXchange Consortium through the PRIDE partner repository (PXD029487).

### 2.3. Subcellular Localization and Functional Classification

**Figure 3:**
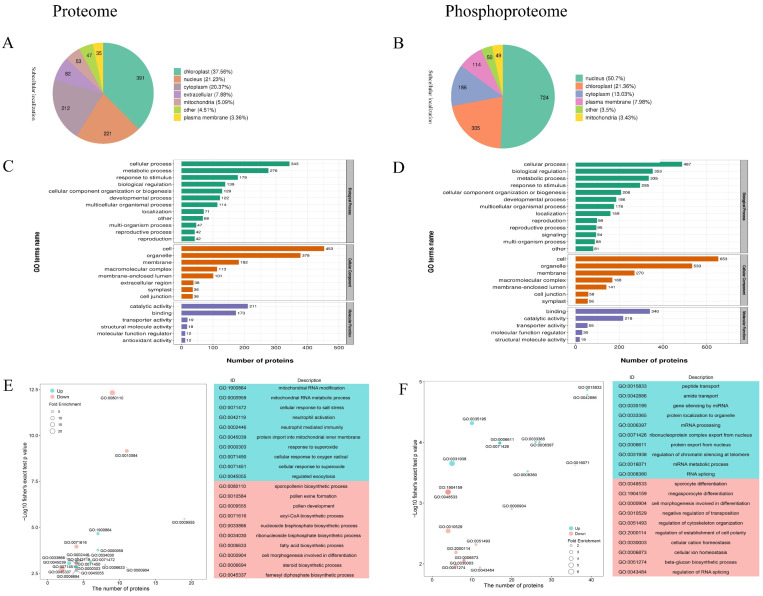
Analysis of the proteome and phosphoproteome data. Subcellular localization prediction and classification statistics of differentially expressed (**A**) proteins and (**B**) phosphosites. GO enrichment analysis of differentially expressed identified enrichment among categories of BP, MF, CC in the proteome (**C**) and phosphoproteome data (**D**). GO-BP classification was analyzed for differential protein (**E**) and phosphoprotein (**F**) enrichment. The pathway names are shown along the vertical axis, the horizontal axis represents the enrichment factor, the sizes of dots in the pathway represent the number of DAPs, and the *p*-value is reflected by the color of each dot.

The subcellular distribution patterns were similar between the proteome and phosphoproteome datasets, with relatively high localization to the nucleus, chloroplasts, and cytoplasm (Figure 3).

We analyzed the differentially abundant proteins (DAPs) and phosphoproteins (DAPPs) enriched in each of the three categories in the gene ontology (GO) classification. The enriched biological processes in the DAPs and DAPPs included the cellular process, metabolic process, and response to stimulus. For cellular components, enriched terms mainly included cells, organelles, and membranes. The enriched molecular function terms mainly included catalytic activity, binding, and transport activity. These results may represent the metabolic or physiological biases of anthers during the Bns under the expression of male sterility. During the enriched biological process (BP) in the differentially expressed proteome and phosphoproteome data, pollen exine formation and sporocyte differentiation were the significantly down-regulated terms. The synthesis and transport of sporopollenin and the development of pollen exine structure are crucial to wheat fertility [21,22]. Sterile rice mutants lack sufficient sporopollenin to deposit exine, and thus pollen walls cannot be thickened to a normal extent [23].

To broaden our analysis (Supplementary Materials Figure S5), we characterized which KEGG pathways were most altered in YS3038 (at the levels of proteome and phosphoproteome) by KEGG enrichment analysis and found that lipid-metabolism-related pathways were highlighted. Of these, starch and sucrose metabolism was down-regulated, while oxidative phosphorylation (OxPhos) was significantly up-regulated at the proteome and phosphoproteome levels. These results indicated that energy metabolism plays an important role in male sterility.

### 2.4. Differentially Expressed Proteins Highlight Fatty-Acid-Related Metabolic Pathways

**Figure 4:**
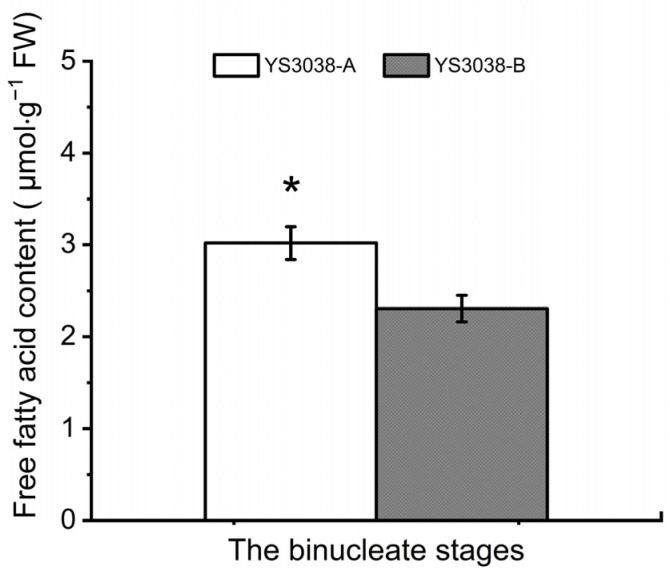
Free fatty acid content in YS3038-A and YS3038-B anthers. Data represent the mean and standard deviation of three replicates. Significant differences were determined by Student’s *t*-test (* *p* < 0.05).

DAPs and DAPPs were enriched and significantly down-regulated in the fatty acid biosynthesis, elongation and the cutin, suberine and wax biosynthesis pathways (Supplementary Materials Table S4). The cutin, suberine, and wax biosynthesis pathways are critical for the normal development of anthers at an early uninucleate stage in the P-type CMS of wheat [20]. The diffusion of cutin and wax onto the surface of anther cell walls is essential for maintaining pollen morphology [23]. In order to confirm the abnormal fatty acid metabolism of YS3038-A, the free fatty acid contents of both YS3038-A and -B were determined (Figure 4). The content of free fatty acids in YS3038-A anthers was significantly higher. Wu et al. showed that the increased contents of free fatty acids in sterile anthers are mainly comprised of C14:0 and C18:1 [24]. The present results showed that excessive free fatty acids in YS3038-A inhibited fatty acid biosynthesis by down-regulating protein expression through feedback regulation.

### 2.5. Abnormal Carbohydrate Metabolism in YS3038-A Anthers

There were 24 DAPPs (with 61 phosphosites) and 53 DAPs enriched in the carbohydrate and energy metabolism pathways, most of which were significantly down-regulated (Supplementary Materials Table S5).

**Figure 5:**
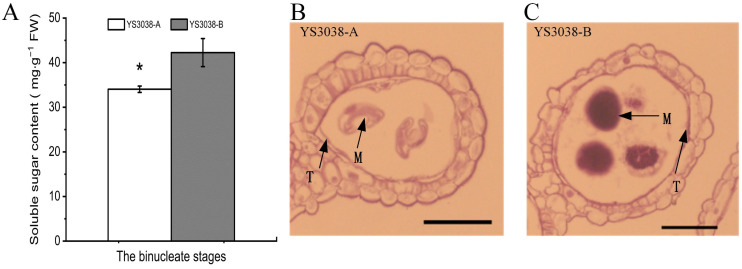
Sugar composition in YS3038-A and YS3038-B anthers. (**A**) Soluble sugar content. PAS staining of anther cross-sections in (**B**) YS3038-A plants compared with those of the (**C**) YS3038-B plants. T, tapetal layer; M, microspores; bars = 200 μm. Significant differences were determined by Student’s *t*-test (* *p* < 0.05).

The above protein expression patterns suggest that YS3038-A male sterility may be caused by the abnormal accumulation of carbohydrates and starch synthesis. Soluble sugar is a key osmoregulation substance, and its normal accumulation can prevent protoplast dehydration and maintain cell resistance to environmental changes [24]. We found that compared with YS3038-B, the total soluble sugar content in YS3038-A anthers was also significantly reduced at the Bns (Figure 5). In addition, the Bns YS3038-A/B anthers were stained with periodic acid-Schiff (PAS). At the Bns, weak positive staining was detected in the tapetum and microspore of YS3038-A anthers, while strong positive staining was detected in the tapetum and microspore of YS3038-B anther. The protein expression pattern in the carbohydrate metabolism pathway reduced the accumulation of total sugar in YS3038-A. Abnormal starch synthesis in pollen led to abortion, which also supported the TMT results.

### 2.6. DAPs and DAPPS Interfere with the Electron Transport Chain, Reactive Oxygen Species Accumulation, and ATP Synthesis

**Figure 6:**
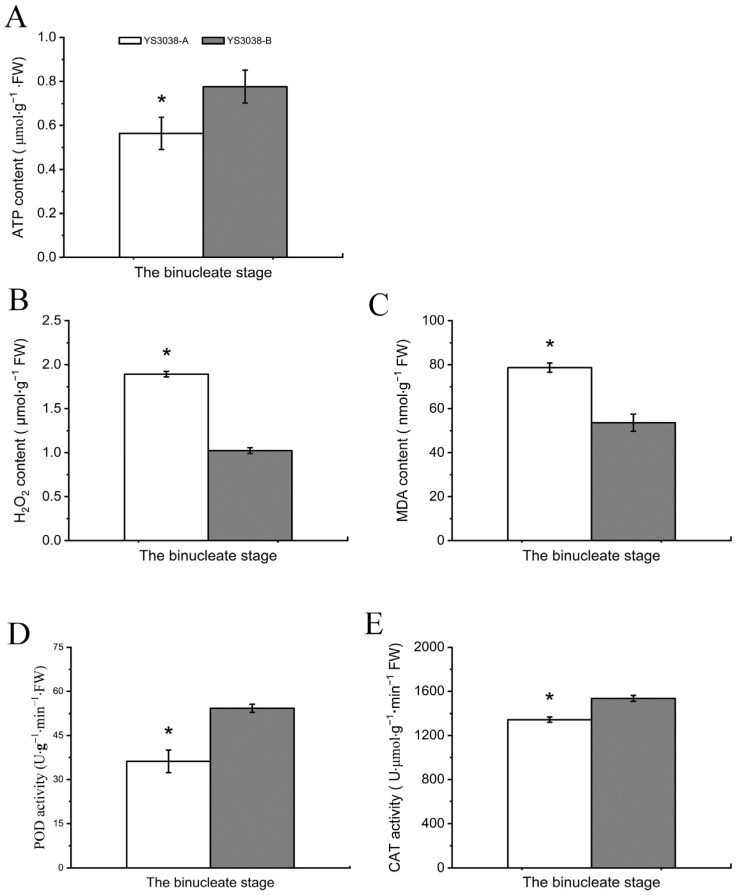
Physiological differences in anther between YS3038-A and YS3038-B. (**A**) ATP contents of anthers. The (**B**) H_2_O_2_ and (**C**) malonaldehyde (MDA) contents and activities of (**D**) peroxidase (POD) and (**E**) catalase (CAT) in anthers. Significant differences were determined by Student’s *t*-test (* *p* < 0.05).

The OxPhos metabolic pathway was enriched for DAPs and DAPPs that were up-regulated (including 10 phosphosites) (Supplementary Materials Table S6). The mitochondrial respiratory chain plays a central role in energy metabolism [25]. Mild continuous oxidative stress increases respiratory electron transport and enhances electron flow to increase the generation of oxygen free radicals, which leads to increases in reactive oxygen species (ROS) generation and the amplification of oxidative stress. The accumulation of hydrogen peroxide induces cell death processes dependent on active cell metabolism [26]. The electron transport chain (ETC) regulates the respiratory rate mainly through the ADP:ATP ratio, which reflects cell energy demand [27]. In order to verify the above results, we measured the total amount of ATP in the binucleate anthers of YS3038-A/B (Figure 6). During the Bns, the ATP level of YS3038-A was low, indicating that YS3038-A might have an insufficient energy supply.

ROS is an important product of oxidative stress in plant cells and a key element of programmed cell death. Under normal circumstances, the ROS system is in a state of dynamic equilibrium [28]. ROS-related indicators in anthers were analyzed to evaluate whether YS3038-A infertility is mediated by the ROS system. The content of H_2_O_2_ produced by YS3038-A in the Bns was higher, and the degree of lipid peroxidation in the cell membrane was increased, which increased the MDA level. Accordingly, the cells experienced substantial nutrient loss, rending them unable to meet the normal development of anthers and resulting in anther abortion. The activities of POD and CAT were also assayed, revealing that the protective enzyme activity in YS3038-A was low in the Bns. These results indicated that the accumulation rate of ROS in YS3038-A was higher than the scavenging rate, and the dynamic balance between the accumulation and scavenging of ROS was abnormal. Studies have shown that excessive ROS accumulation can lead to abnormal development of anther apoptosis and ultimately lead to male sterility [28,29,30].

### 2.7. Phosphoproteomics Reveals the Protein Kinase Regulatory Network of YS3038

**Figure 7:**
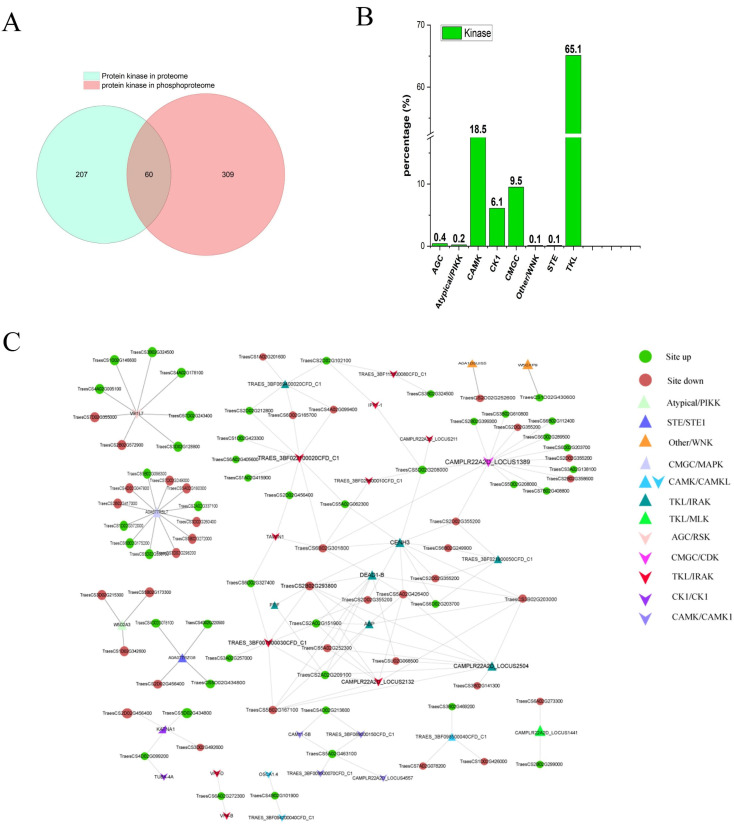
Overview of the kinases identified in phosphoproteome. (**A**) Overlap of the kinases identified in our datasets. (**B**) Significantly enriched kinase families in the phosphoproteome were identified. (**C**) The kinase–substrate network was extracted from the phosphoproteome data. Triangles represent up-regulated kinases, V-shapes represent down-regulated kinases, and circles represent substrates.

Protein kinases (PKs) are found in almost all plant organs, where they enable plants to adapt to changing environments [11], and many studies suggested that PKs affect the male reproductive development of plants [12]. Here, we identified 267 and 369 PKs in the proteome and phosphoproteome (Figure 7A), respectively, with 790 phosphosites identified. Nearly half of the PKs have multiple phosphosites (Supplementary Materials Table S7). This indicated that phosphorylation is widely present across kinases involved in anther development in YS3038.

PKs interact with each other by phosphorylation. Different kinases preferentially phosphorylate specific substrates with conserved sequence motifs [12]. In total, 233 kinase–substrates pairs were found among the phosphoproteins in our study, and the interaction network was visualized using Cytoscape (Figure 7 and Supplementary Materials Table S8). In order to predict the relationship between phosphosites and their corresponding kinases and determine how these relationships mediate fertility, the identified PKs were classified according to their phylogenetic tree into AGC/RSK, TKL/IRAK, STE/STE11, other/WNK, CMGC/CDK, CMGC/MAPK, CK1/CK1, CAMK/CAMK1, and atypical/PIKK groups. In these regulatory networks, a total of 40 kinases were identified, corresponding to 166 substrates of 233 phosphosites. TKL kinases were the largest kinase family identified, with 21 kinases, accounting for 52.5% of all kinases, followed by CAMK (7, 17.5%). This finding suggested that kinases play a key role in the regulation of fertility in YS3038. CK1 and CMGC families were represented at higher percentages than other families, implying that these kinases may play particularly important roles in YS3038 fertility alteration.

### 2.8. Other Sterility Candidate Proteins Identified among DAPs and DAPPs

**Figure 8:**
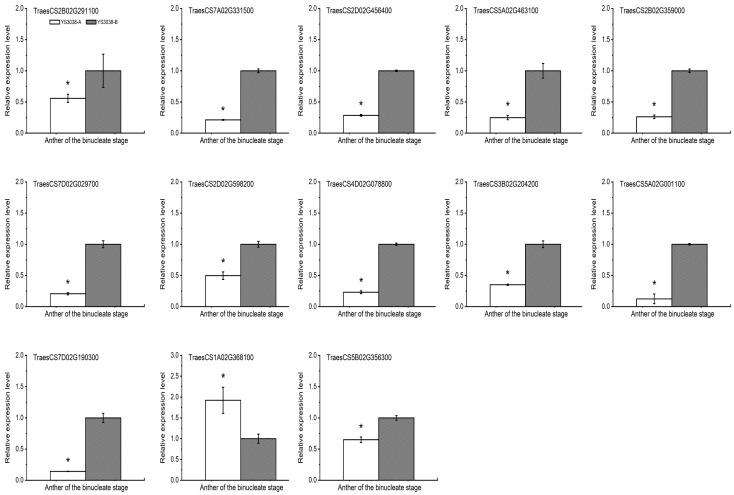
Transcriptional expression patterns of selected differentially expressed protein. Significant differences were determined by Student’s *t*-test (* *p* < 0.05).

Some homologs of rice proteins related to male sterility were found among the identified DAPs/DAPPs by amino acid sequence alignment (Supplementary Materials Table S9). The genes encoding the proteins identified in the proteome and phosphoproteomic studies (listed in Supplementary Materials Table S9) were selected to validate the dynamic transcriptional expression patterns of these representative genes by qRT-PCR (Figure 8). However, the expression patterns of three genes (*TraesCS4D02G078800*, *TraesCS3B02G204200*, *TraesCS7D02G190300*) were the opposite of their protein expression patterns. Previous studies indicated that the results obtained by proteomic analysis do not necessarily agree with the results of transcriptional analyses [8]. The differences in these three proteins might be explained by post-translational regulation [31]. Additionally, 12 genes were down-regulated at the binucleate stage. The significant decrease in the expression of these genes during the binucleate stage suggested that these proteins may be associated with male sterility in YS3038. Remarkably, the significant down-regulation of the above key proteins (TraesCS6D02G238700 and TraesCS6B02G255100) involved in lipid metabolism at the transcription and translation levels may be the cause of abnormal pollen development and defective anther dehiscence in YS3038. This result supports the above results regarding the role of abnormal lipid metabolism. The low expression of carbohydrate transporters may also contribute to YS3038-A sterility. In our results, the homologs of OsUgp1 (a UDP-glucose pyrophosphorylase, OsUgp1 is mainly involved in callose deposition and carbohydrate metabolism in pollen mother cells and meiosis stages) and OsMST5 (an energy-dependent monosaccharide transporter, OsMST5 participates in pollen germination and microspore energy supply to support pollen tube growth) were down-regulated at both the transcriptional and translational levels. The abnormal expression of these proteins could cause abnormal starch and wax metabolism in YS3038-A, thus aborting pollen. All of these results elucidate and guide the continued study of male sterility in wheat.

Pollen development is a highly specific process that involves signal transduction, cell cycle control, and programmed cell death (PCD). TaPDCD5 (TraesCS1A02G368100) expression was up-regulated, and the apoptosis inhibitor protein TaAPI5 (TraesCS6D02G180700) was down-regulated; their regulatory patterns were consistent at both the translational and transcriptional levels. The two proteins are homologs of OsPDCD5 (*OsPDCD5* is homologous to mammalian PCD gene 5; the decreased expression of *OsPDCD5* induces pollen sterility in rice) and OsAPI5 (*OsAPI5* encodes a putative homolog of antiapoptosis protein Api5 in animals and results in the delayed degeneration of the tapetum, leading to defects in the formation of male gametophytes), respectively. Pollen abortion in most CMS rice lines is associated with abnormal tapetum degradation resulting from a misregulation of the PCD pathway; pollen development was sensitive to the observed transcript abundances of *OsPDCD5* and *OsAPI5* [32,33].

### 2.9. Functional Verification of TaPDCD5 via BSMV-VIGS

**Figure 9:**
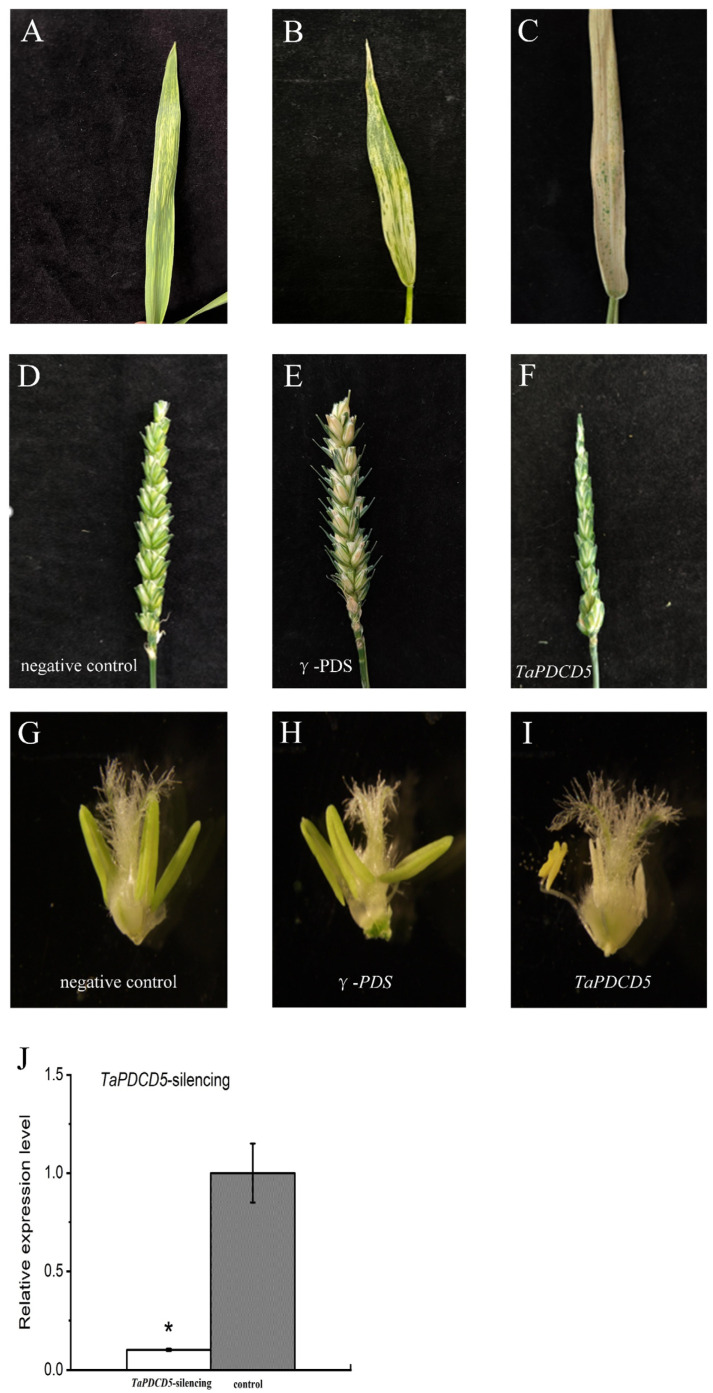
Functional verification of *TaPDCD5* via BSMV-VIGS. (**A**) Leaf characteristics of positive control (*PDS* gene-silenced) plants. (**A**), Early stage of positive control plants. (**B**), Metaphase of positive control plants. (**C**), Late stage of positive control plants. (**D**–**F**) Fertility of ears. (**D**), Negative control. E, Positive control. F, *TaPDCD5*-silenced plants. (**G**–**I**) Anther morphology. (**G**), Negative control. (**H**), Positive control. L, *TaPDCD5*-silenced plants. (**J**) Relative expression of gene-silenced plants. Significant differences were determined by Student’s *t*-test (* *p* < 0.05).

The γ empty vector was used as the negative control, and the phytoene desaturase (*PDS)* gene was used as the positive control (virus-induced PDS gene silencing results in visible leaf photo-bleaching). The positive control group showed an obvious whitening of leaves. The silencing of the *TaPDCD5 (TraesCS1A02G368100)* led to a significant decrease in the expression level of *TaPDCD5* and seed setting rate of YS3038 under fertile conditions (Figure 9 and Supplementary Materials Table S10). In the *TaPDCD5*-silencing plant, the anther showed an abnormal shape. YS3038 anther development was sensitive to the transcript abundance of *PDCD5*. It was reported that *PDCD5* was highly expressed in old organs [34], The antisense expression method down-regulates the expression of *PDCD5*, resulting in male sterility in photoperiod-sensitive rice varieties [32]. Furthermore, control of the protein abundance of *TaPDCD5* by natural or artificial alteration of environmental conditions may significantly affect wheat fertility.

## 3. Discussion

### 3.1. A regulatory Network of Energy Metabolism a Candidate for TCMS in Wheat

**Figure 10:**
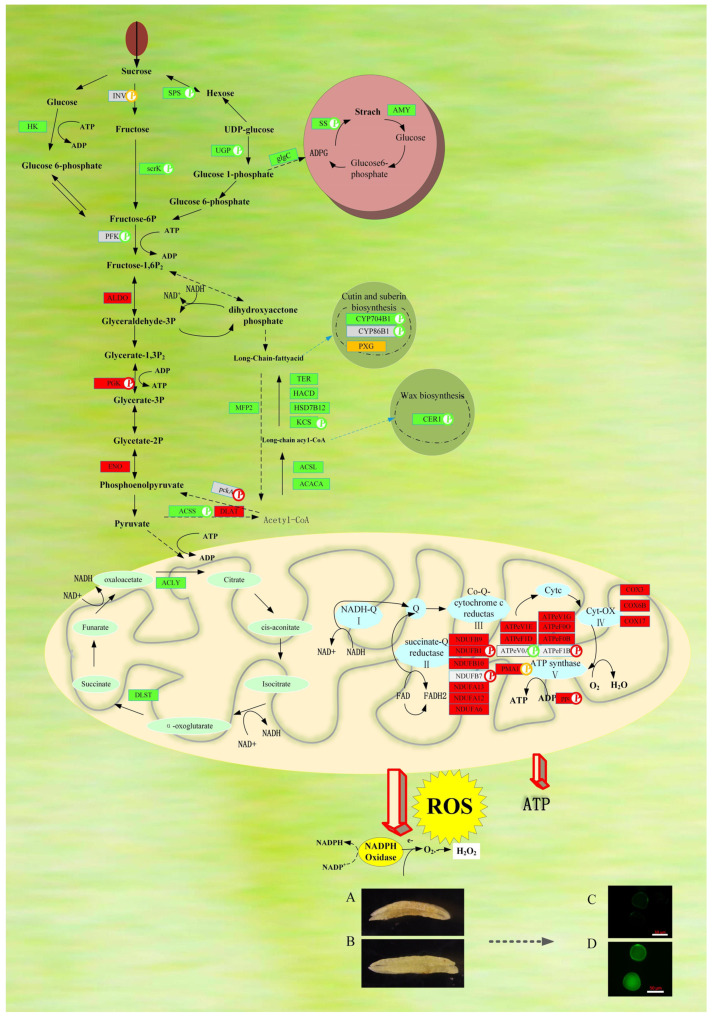
Schematic overview of the metabolic pathways associated with the differentially expressed proteins identified in pollen abortion of YS3038. DAB staining of (**A**) YS3038-A and (**B**) TabYS3038-B. FDA staining of (**C**) YS3038-A and (**D**) YS3038-B. Figure is drawn by me using Visio according to our experimental results on the basis Supplementary Materials Tables S4–S6 in article of Wang et al. (2019) [29]. Protein names in gray indicate they were not observed in our proteomic dataset, P in a circle indicates phosphoproteins, green indicates down-regulation, red indicates up-regulation, yellow indicates both up- and down-regulation.

To identify the biochemical pathways related to DAPs and DAPPs between male sterile YS3038-A and male fertile YS3038-B, a proteomic analysis was conducted. Among the many results, we found that fructokinase (scrK) and hexokinase (HK) were significantly down-regulated in YS3038-A anthers, and 6-phosphofructokinase (PFK) had a significantly down-regulated phosphosite (Figure 10 and Supplementary Materials Table S5). The regulation pathway of HK and PFK is an irreversible step at an important node in glycolysis [8]. The HK catalytic reaction initiates all hexose utilization pathways, including leading the cleavage products of sucrose into the starch biosynthesis pathway, and the abnormal expression of HK activity can lead to male sterility [30,35]. The expression of fructose-bisphosphate aldolase (ALDO), phosphoglycerate kinase (PGK), and enolase (ENO) in the glycolysis/gluconeogenesis reversible pathway was up-regulated, suggesting that a lack of soluble sugar in YS3038-A anthers led to the activation of the gluconeogenesis pathway. In addition, the protein expression levels of sucrose-phosphate synthase (SPS), UTP-glucose-1-phosphate uridylyltransferase (UGP), glucose-1-phosphate adenylyltransferase (glgC), sucrose synthase (SS), and α-amylase (AMY) in the starch synthesis pathway were significantly down-regulated. The down-regulation of the abundance of SPS and SS may cause abnormal starch accumulation [29]. Abnormal starch metabolism might be an important factor in the binucleate microspore abortion [20]. UGP is mainly involved in callose deposition and carbohydrate metabolism in pollen mother cells and during meiosis; OsUgp2 was observed in binucleate pollen [36,37]. A reduced expression of beta-fructofuranosidase (INV) can enhance trends in sucrose content [38]. INV had two abnormally expressed phosphosites, SPS contained four significantly down-regulated phosphosites, and UGP had a down-regulated phosphosite. These phosphosites may play a role in sterility mediated by TCMS.

There is growing evidence that the pollen wall is mainly composed of sporopollenin, and lipid metabolism is essential in the biosynthesis of sporopollenin [24,39]. Down-regulated proteins and phosphoproteins were found to be enriched in fatty acid biosynthesis, the elongation pathway and the cutin, suberine and wax biosynthesis pathways (Supplementary Materials Table S4). *CYP704B1(TaMS26)* encodes a cytochrome P450 mono-oxygenase, playing an important role in the hydroxylation of the fatty acid constituents of the sporopollenin precursors and affecting the deposition of sporopollenin in the tapetum and microspores [21,40]. These metabolic events affect the formation of the YS3038-A pollen wall, resulting in sterility.

Pyruvic acid is the key node of the energy metabolism pathway network, after which the decarboxylation of pyruvic acid by acetyl-CoA can occur [24,29]. The feedback of the excessive accumulation of free fatty acids in YS3038-A anthers likely inhibited protein expression in the fatty acid synthesis pathway. The up-regulated expression of pyruvate dehydrogenase (DLAT) may increase the accumulation of acetyl-CoA. It is hypothesized that the accumulation of acetyl-CoA in YS3038-A mainly flows to the fatty acid metabolism pathway, and feedback regulates the down-regulation of acetyl-CoA synthetase (ACSS) protein levels. As a substrate of gluconeogenesis, acetyl-CoA regulates the up-regulation of a protein phosphosite in phosphoenolpyruvate carboxykinase (pckA). In addition, inhibition of the TCA pathway is an important cause of anther male sterility. Two TCA cycle-related proteins, citrate synthase (ACLY) and 2-oxoglutarate dehydrogenase (DLST), were down-regulated in YS3038-A anthers.

NAD (P) H and ATP are necessary for the main energy consumption reactions in cells during pollen development [41] and the down-regulation of hexokinase promotes ATP production through the OXPHOS system [42]. The insufficient ATP content in YS3038-A may be an important cause of dysfunction in the OxPhos pathway. The up-regulated proteins in YS3038-A were mainly concentrated in complex II, complex IV, and the ATP synthase complex (Supplementary Materials Table S6). Complex II directly links the TCA and ETC, and the increased activity of complex II is accompanied by increased ROS production [26]. In complex II, six subunits were highly expressed, and two subunits were phosphorylated. Complex IV is also critical for ROS production [25]. Three subunits of cytochrome oxidase were up-regulated, and ATP synthase was found to play a role in PCD [27]. Increased ATP demand during temperature stress induces the over-expression of specific ATP synthase subunits. The increase and decrease in the abundance of some F-ATPase subunits may depend on species, duration, and severity of stress. For example, the expression of ATP synthase subunits in low-temperature-sensitive sunflower varieties can increase, and ATP synthase participates in the regulation of CMS fertility through an unknown mechanism [43]. The abnormal expression of these ATP synthase subunits in YS3038-A (accompanied by differential phosphorylation of phosphosites) may lead to the dysfunction of ATP synthase, affect mitochondrial energy output, and induce changes in mitochondrial membrane potential, thereby aggravating microspore abortion. The oxidative stress of YS3038-A associated with electron transport in mitochondria could result in the amplification of hydrogen peroxide production (Figure 10A,B), ATP loss, and ultimately dysfunctional PCD.

According to these results, we suggest that the energy demand is not met during the continuous low-temperature stimulation, disturbed carbohydrate and lipid metabolism stimulates ETC to accumulate ROS in pollen development, thereby initiating PCD and pollen abortion in the YS3038-A anthers (Figure 10C,D).

### 3.2. Protein Kinase-Related DAPs and DAPPs

Calcium-dependent protein kinases have critical functions in regulating plant growth and development. Unexpectedly, 16 calcium-dependent protein kinases with 28 phosphosites are homologous to OsCPK25 in our database (Supplementary Materials Table S7). OsCPK25 is involved in the regulation of stamen development and maintains the normal number of stamens in rice [44]. Six casein kinase I homologs of rice EL1 were phosphorylated, including eight phosphosites. The enhanced response of el1 mutants to GA signal induces the overexpression of CYP703A3 and KAR genes related to GAMYB and pollen formation during spikelet development, resulting in defective anther development and pollen viability [45]. These two RLKs (DRUS1 and DRUS2) inhibit protease-mediated cell degradation. The late development of anthers affects the biosynthesis of starch in pollen [46]. We identified a DRUS2 homolog in the phosphoproteomic analysis. In addition, the mitogen-activated protein kinase (MAPK) cascade is an evolutionary conserved signal module, which converts environmental and developmental signals into a series of cellular responses. Seven MAPKs containing 10 phosphosites were identified in the phosphorylation data set, and they were homologous to OsMPK6 and DSG1, which encodes OsMAPK6; the anthers of dsg1 mutants became smaller, suggesting their involvement in anther development [47]. These previously studied kinases, which are homologous to our identified kinases, may provide evidence for future candidate wheat sterility genes.

### 3.3. Transcription Factors

It is well-established that transcription factors (TFs) play important roles in anther and pollen development [48]. However, whether and how TF activity is controlled by protein modification in TCMS wheat has not been widely examined. In our study, 253 TFs belonging to 36 different families were identified based on the alignment of our proteomic and phosphoproteomic data with wheat TFs in PlantTFDB (Supplementary Materials Figure S6 and Table S11). Protein phosphorylation is widely involved in the modification of transcription factors, which may regulate their transcriptional activity in fertility conversion. Some protein families have been identified as having more phosphosites, including C3H, bZIP, bHLH, and MYB. This means that their family members may act as similar regulatory factors, regulating the fertility of YS3038 anthers through phosphorylation modification.

Members of some TF families were determined to have significant effects on anther and pollen development in rice and/or Arabidopsis, such as ARF, MADS, SBP, and bHLH [48]. *TaAGL6* (MADS-box gene) was found to be very important for floral organ and spikelet meristem development [49]. Our proteomic and phosphoproteomic datasets revealed six homologs of known fertility-related proteins in rice. *OsLBD12-1* [50], *OsMYB80/MYB103* [51], and *OsMS1* (which encodes PHD-finger protein) [52] are essential for tapetum development and pollen formation. Notably, we also found that these TFs homologues are phosphorylated and have multiple phosphosites, which may indicate that phosphosites differ in their effects on anther development. These findings, together with the discovery of TFs homologs that were determined to regulate fertility, may clarify the mechanism by which sterility is regulated in YS3038.

### 3.4. Epigenetic Differences

TCMS in wheat has typical characteristics of epigenetic systems. Epigenetic modifications can affect gene expression by changing the chromatin state without alteration of the DNA sequence [30]. Epigenetic modification, characterized by DNA methylation, histone modification, and chromatin remodeling, is an important regulator of anther development. Abnormal epigenetic modification can disorder anther development, leading to male sterility [9,12]. In our datasets, 95 putative epigenetic phosphoproteins (216 phosphosites) were identified (Supplementary Materials Table S12).

Dicer, RNA-dependent RNA polymerase (RDR), and Argonaute (AGO) proteins are the core components that induce gene silencing and also participate in the initiation and maintenance of RNA molecules, which is at the core of RNAi processes. OsDCLs, OsRDRs, and OsAGOs show specific or preferential expression patterns during reproductive development, which might involve reproductive-development-specific gene regulation mechanisms [53,54]. Fifteen RDRs and ten AGOs phosphoproteins were identified during the Bns in YS3038. As a member of the AGO family, MEL1 regulates the meiosis process of meiotic cells through epigenetic effects [55]. OsAGO2 is highly expressed in anthers; after knocking out OsAGO2, tapetum degeneration and pollen abortion are initiated earlier [30]. Our results identified four DAPPs that are OsAGO homologs.

Forty-seven identified phosphoproteins (83 phosphosites) were related to histones, which are the substrates of many enzymes. Histone modification can control specific biological functions [56]. Our datasets identified 23 histones (29 phosphosites), providing new evidence for histone modification in anthers. Histone modification markers are recognized by specific chromatin proteins, with the remodeling of chromatin structures regulating DNA accessibility transcription and other activities [57]. A progressive loss of fertility has been observed in some independent antisense OsDDM1(CHR746) lines, leading to sterility [57]. The proteins related to histone methylation are phosphorylated in association with YS3038 fertility alteration, and a determination of whether they participate in fertility regulation requires further study.

In addition, we identified RNA-directed DNA methylation (RdDm) phosphoprotein. RdDM is an important regulatory event involved in epigenetic modification, which can trigger transcriptional gene silencing. The protein phosphorylation of factors in the RdDM pathway in wheat has not been reported previously. RdDM may be involved in the regulation of LDMAR expression, thus inducing the fertility conversion of photo-sensitive sterile rice [58]. Phenotypic changes caused by epigenetic modification are affected by the environment, and their regulatory mechanisms are complex, which also enables epigenetic modification to more precisely regulate the expression of plant genes.

## 4. Materials and Methods

### 4.1. Plant Materials

*Triticum aestivum* L. TCMS line YS3038 was first isolated from among hybrid offspring by Professor Beiru He in 2003, and it is preserved in our laboratory (College of Agronomy, Northwest A&F University, Yangling, China). YS3038 seeds were sown in an experimental field of Northwest A&F University on 6 October 2020. After the regeneration period in the spring of 2021, YS3038 seedlings were transplanted into glass greenhouses (60% relative humidity). A uniform growth environment was maintained for the wheat before booting. One week before the booting stage (https://bookstore.ksre.ksu.edu/pubs/MF3300.pdf, accessed on 6 March 2021), plants were moved to different fertility temperatures, a greenhouse with either a cooler sterile (YS3038-A) environment (17/13 ℃) or a warmer fertile (YS3038-B) environment (24/20 ℃). After three biological replicates were subjected to 4′,6-diamidino-2-phenylindole (DAPI) staining and subsequent electron microscopy, samples of anthers collected at the binucleate stage were quickly placed in liquid nitrogen and then stored in an ultra-cryogenic freezer (−80 °C).

### 4.2. Total Protein Extraction and Trypsin Digestion

A sample of each anther for analysis was retrieved from −80 ℃ refrigeration, and the appropriate amount of each sample was added to liquid nitrogen in a pre-cooled grinding tube and fully ground into powder. For each sample, four times the volume of phenol extraction buffer was added, followed by ultrasonication for 35 min. Equal volumes of Tris-balanced phenol were added to the extract, and the samples were centrifuged at 10,000× *g* and 4 °C for 10 min. The supernatant was collected, and five times the volume of 0.1 M ammonium acetate in methanol was added prior to precipitation overnight. Protein precipitates were washed with methanol and acetone with 8 M urea re-dissolved, and a BCA kit was used for protein concentration determination.

The anther protein samples were added to equal amounts for enzymatic solution, and the volumes were adjusted with lysate. Then, TCA was slowly added until a 20% final concentration was reached, before mixing for 2 h (4 ℃). Then, samples were centrifuged at 10,000× *g* and 4 °C for 5 min, washed, and received additions of pre-chilled acetone three times, being allowed to settle after each addition. Dry precipitates were formed after the addition of tetraethylammonium bromide, and ultrasonication was used to break up the precipitate after adding trypsin, after which the enzymatic solution was allowed to sit overnight. Dithiothreitol was added to reduce samples for 30 min (at 56 ℃). Iodoacetamide was then added, and samples were incubated for 20 min at room temperature.

### 4.3. TMT Labeling and Phosphopeptide Enrichment

The peptide segments with trypsin were vacuum-frozen, dried after desalination with Strata X C18 (Phenomenex, Torrance, CA, USA), and processed according to the manufacturer’s TMT kit protocol. The tryptic peptides were fractionated into fractions by high pH reverse-phase HPLC.

Peptide segments were then dissolved in a concentrated buffer solution, the liquid was collected as IMAC material, and the incubated sample was gently shaken. Finally, elution of peptides, desalting, and vacuum freezing of the extraction were conducted to prepare samples for LC-MS/MS Analysis.

### 4.4. LC-MS/MS Analysis

Peptides were dissolved in phase A (0.1% formic acid and 2% acetonitrile aqueous solution) of the liquid chromatography mobile phase and separated by EASY-nLC 1000 ultra-high performance liquid chromatography. The peptides were separated by ultra-high performance liquid chromatography (UPLC), injected into NSI ion sources for ionization, and then analyzed by QE plus mass spectrometry. Both peptide parent ions and their secondary fragments were detected and analyzed using high-resolution Orbitrap spectrometry. The data acquisition mode used the data-dependent scanning (DDA) program.

### 4.5. Database Search and Data Analysis

Secondary mass spectrometry data were retrieved using the Maxquant search engine (v.1.5.2.8). Fold change (FC) was used to quantify differences (Supplementary Materials Figure S4A), and down-regulated and up-regulated proteins were assessed according to thresholds of *P* ≤ 0.05 and |log_2_ FC| ≥ 1.3. Information about rice proteins was obtained from the China Rice Data Center (https://www.ricedata.cn/, accessed on 1 October 2021). Transcription factors were also identified using the Plant Transcription Factor Database (http://planttfdb.gao-lab.org/, accessed on 3 October 2021). Confirmation of homologous genes between crops was conducted using BLASTp. We confirmed the preliminarily identified protein domain with the NCBI-CDD web server. The Kyoto Encyclopedia of Genes and Genomes (KEGG) database was used for pathway enrichment analysis. The InterPro database was used to analyze the enrichment of functional domains of differentially expressed proteins. The software package MoMo using the motif-x algorithm was utilized to analyze the motif features of the modified sites. With the STRING (v.10.5) protein network interaction database, 2D annotation enrichment was analyzed using Perseus software and visualized using the R statistical programing environment. The differential protein interaction network was then visualized using the Cytoscape platform.

### 4.6. Phenotypic Characterization and Cytological Observations

Anthers were fixed with FAA fixative and stained with DAPI, periodic acid-Schiff (PAS), and fluorescein diacetate (FDA) following previously described methods [29,59]. The FDA assay was performed to assess the vitality of fresh pollen grains. 3,3′-diaminobenzidine (DAB) staining with the diaminobenzidine method was conducted using 1 mL of 10 mM Tris-HCL, DAB, and 0.03% CoCL_2_ mixed, to which 10 mL of 30% H_2_O_2_ was added, after which the solution was mixed again. Anthers were placed in DAB staining solution in dark incubation for 3 h and then placed in deionized water for cleaning. Mature pollen was stained with I_2_-KI and peroxidase (following the aniline method). Images of the microspores were acquired using ICc5 color camera (ZEISS, Oberkochen, Germany) mounted onto a biological fluorescence microscope (ZEISS Imager M2, Oberkochen, Germany). Images of the anther and the seed status after wheat pollination were acquired using a stereoscopic microscope (OLYMPUS SZX16, Tokyo, Japan).

### 4.7. Determination of Physiological Indexes of Anthers

The enzymatic activities of peroxidase (POD) and catalase (CAT) as well as the contents of H_2_O_2_ and malonaldehyde (MDA) contents in anthers were determined following previously published methods [60,61]. The ATP and soluble sugar contents were also determined following previous methods [8]. Determination of the free fatty acids content was conducted as described by Wu et al. [24].

### 4.8. Quantitative Real-Time PCR

Gene-specific primers were designed using Primer 5.0 (Supplementary Materials Table S13). The actin gene of wheat (forward primer, 5′-CTCCCTCACAACAACCGC-3′; reverse primer, 5′-TACCAGGAACTTCCATACCAAC-3′) was used as a reference to normalize the expression levels of the assayed genes. Quantitative real-time PCR (qRT-PCR) analysis was performed as described by Han et al. [17], and we used the 2^−∆∆Ct^ analysis method to determine the relative expression levels.

### 4.9. Functional Verification of Candidate Genes via BSMV-VIGS

A 200-bp fragment of *TaPDCD5* (ID: *TraesCS1A02G368100*) gene was amplified using cDNA as a template. Homologous arm of PacI (TAGCTAGCTGATTAATTAA) and NotI (TTGCTAGCTGAGCGGCCGC) restriction sites were added to the 5′ ends of the forward and reverse primers, respectively, along with protective bases. After the digestion of the plasmids of γ-PDS and T-*TaPDCD5* by PacI and NotI, the vector and the gene (*TaPDCD5*) fragments were connected, and the correct clone was used for subsequent experiments. The γ-PDS and γ-*TaPDCD5* plasmids were digested with BssHII, the α and γ plasmids were digested with MluI, the β plasmid was digested with SpeI. Production of the transfection mixture and viral infection of seedlings was carried out according to Han et al.’s method [62].

## 5. Conclusions

Anther development is one of the most important processes of sexual reproduction in wheat. Our results provide new insights into the formation of the regulatory mechanism of TCMS. Our study revealed the various potential protein and phosphoprotein control networks associated with TCMS, which will be valuable for understanding male sterility in wheat anthers. To identify more potential proteins controlling male sterility in wheat, we also searched for wheat homologs of known proteins causing male sterility in rice. We have provided the first thermosensitive sterile wheat anther phosphoproteomic dataset for use by the broader scientific community. This critical resource supports further research on wheat heterosis utilization and provides an important empirical basis for improved crop breeding in wheat.

## Data Availability

Data obtained in this study were deposited in the ProteomeXchange Consortium through the PRIDE partner repository (PXD029487).

## Supplementary Materials

The following supporting information can be downloaded at: https://www.mdpi.com/article/10.3390/ijms23126428/s1.
