# Supplementary figures and images for: Proteomic and Phosphoproteomic Analyses Reveal a Complex Network Regulating Pollen Abortion and Potential Candidate Proteins in TCMS Wheat
Source: Int J Mol Sci. 2022 Jun 8;23(12):6428. doi: 10.3390/ijms23126428 (PMC9224247; doi:10.3390/ijms23126428)

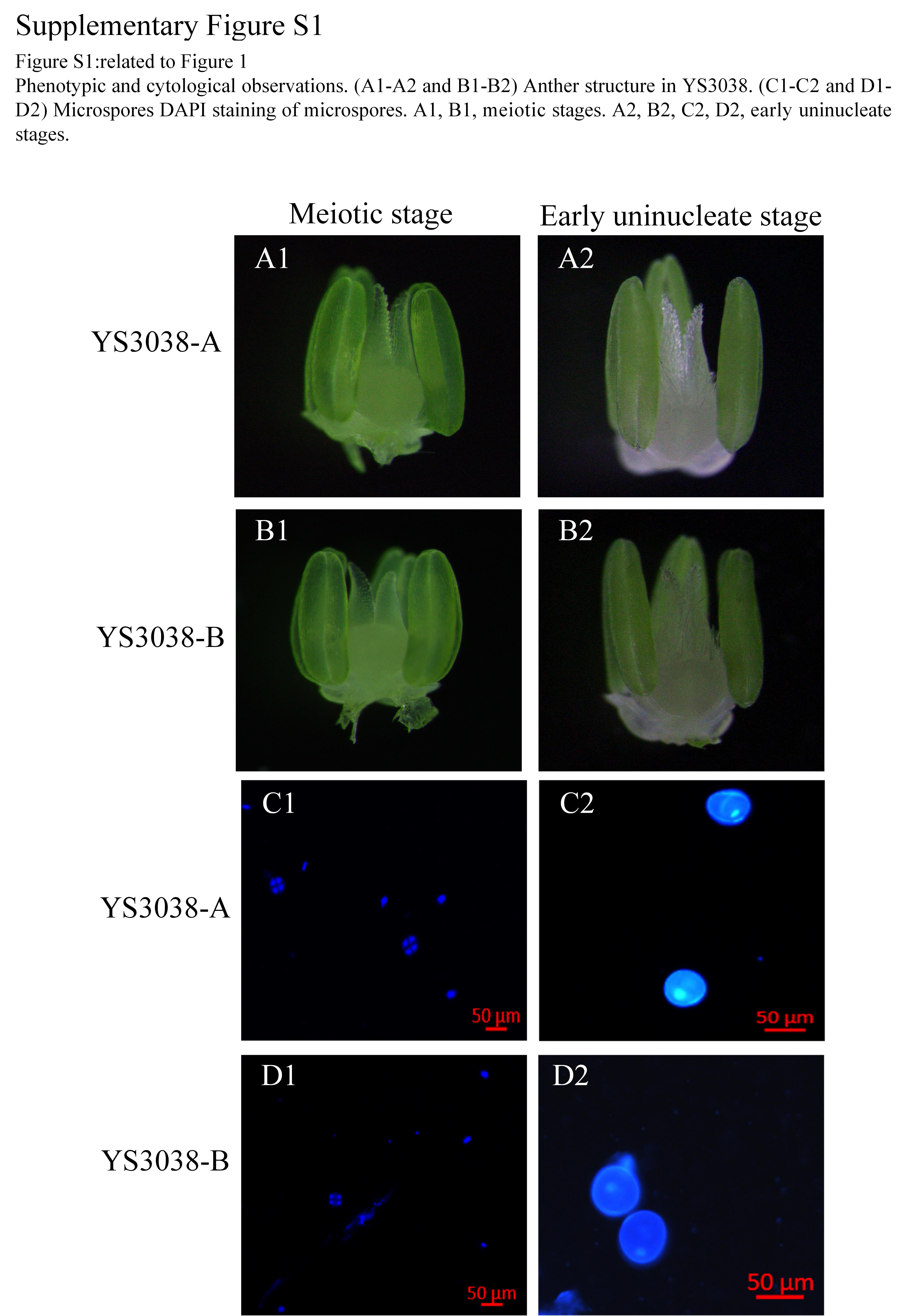

Supplement: Supplementary file 1 [file ijms-23-06428-s001.zip › Supplementary Files/Supplementary Figures/Figure S1.jpg]

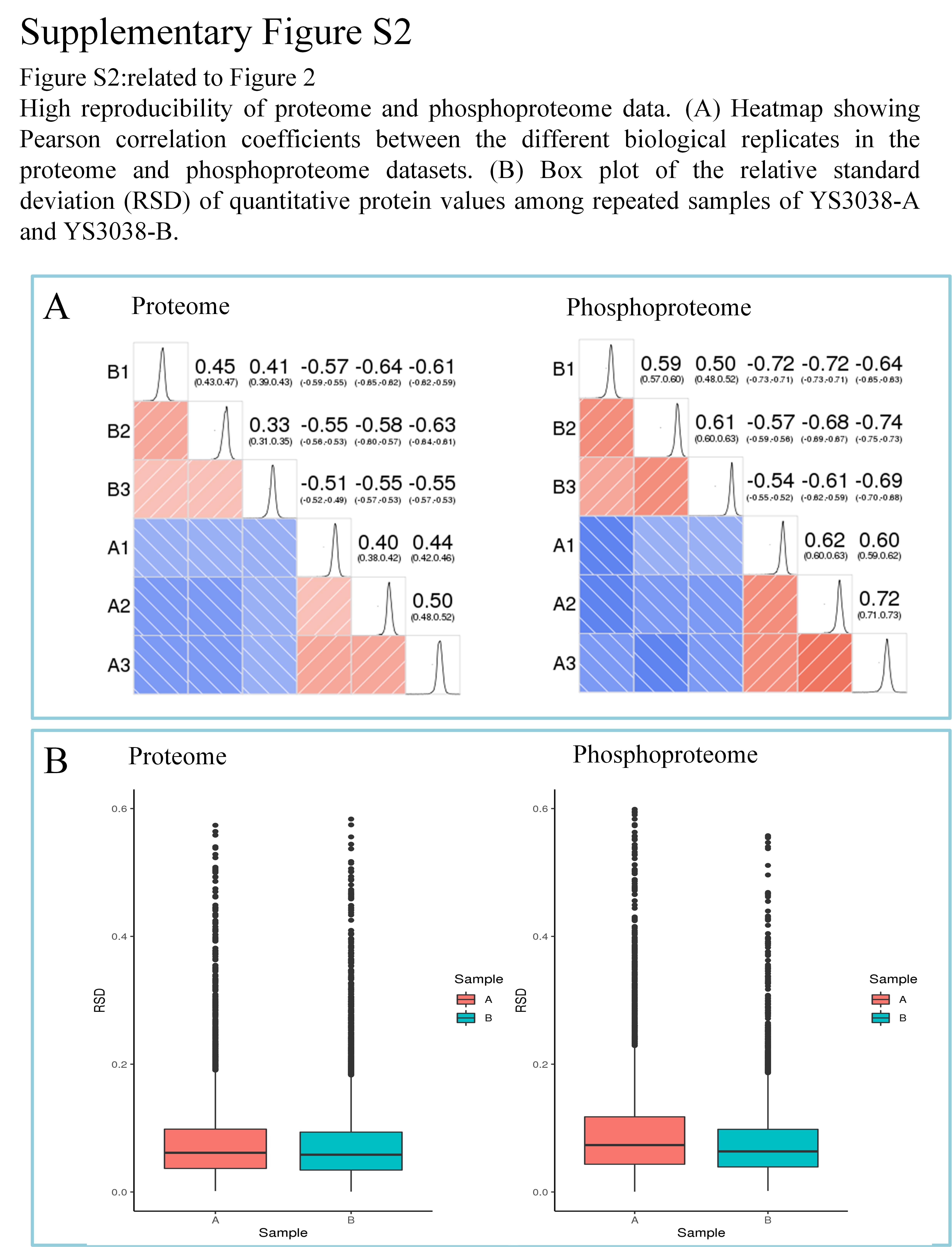

Supplement: Supplementary file 1 [file ijms-23-06428-s001.zip › Supplementary Files/Supplementary Figures/Figure S2.jpg]

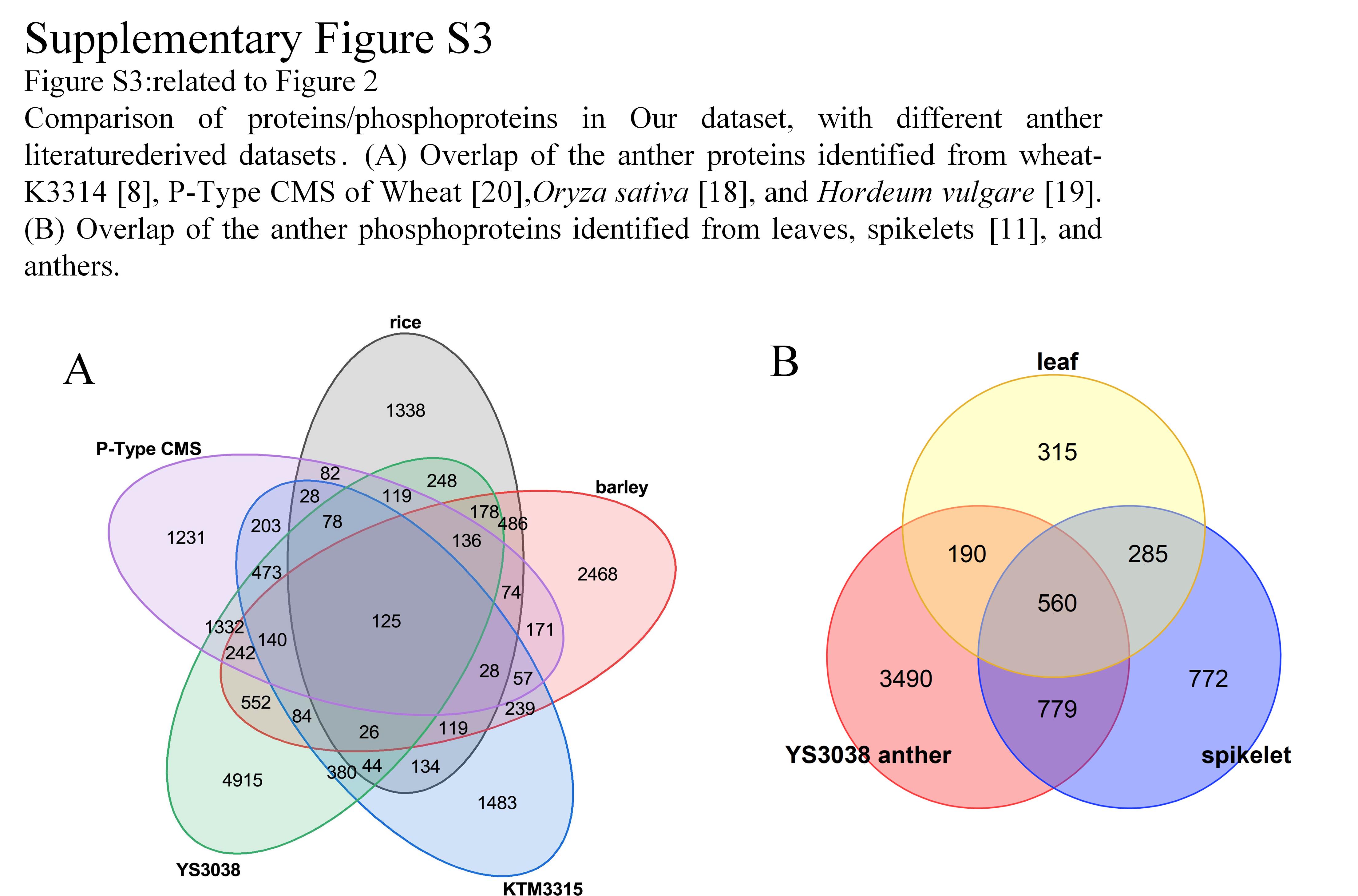

Supplement: Supplementary file 1 [file ijms-23-06428-s001.zip › Supplementary Files/Supplementary Figures/Figure S3.jpg]

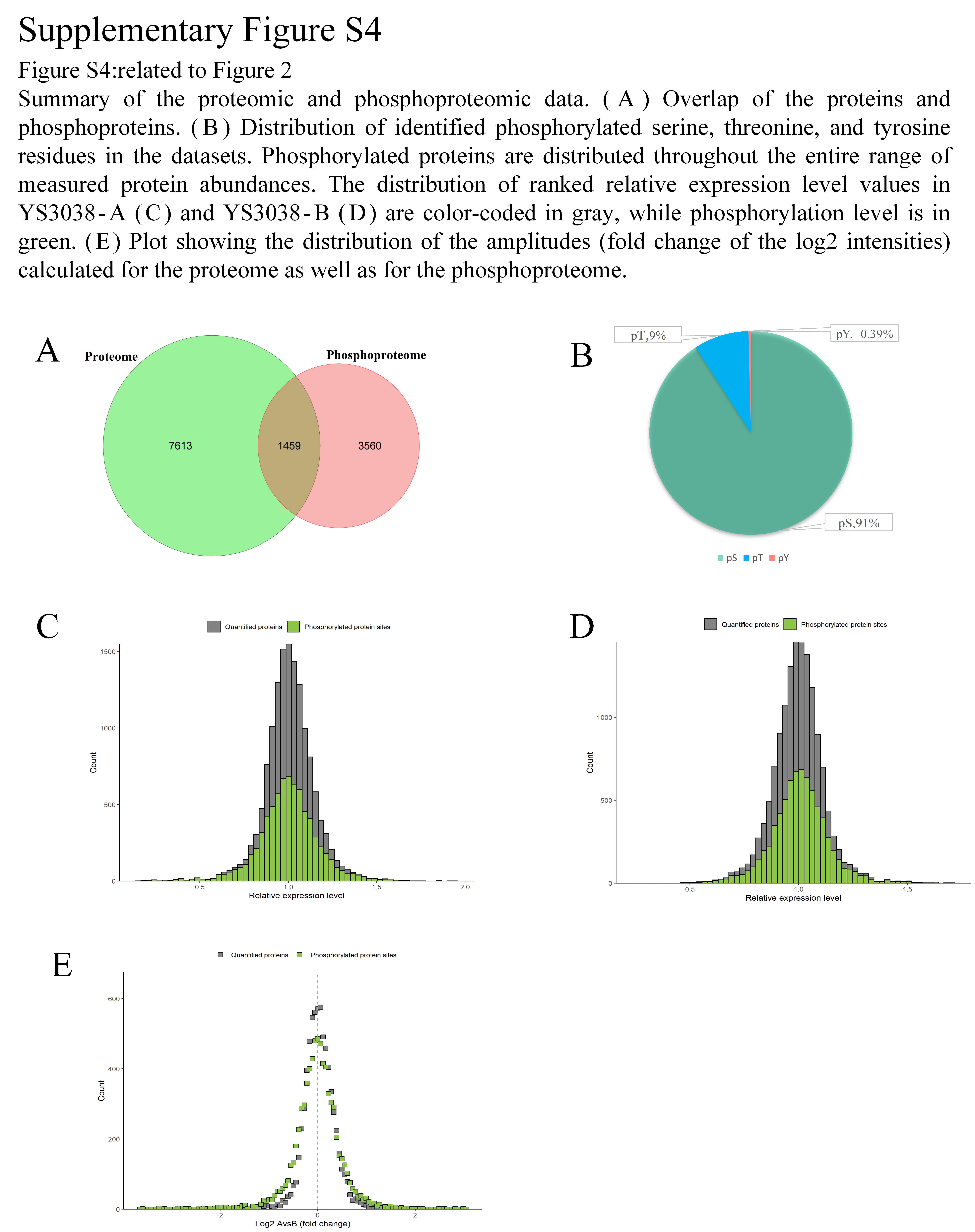

Supplement: Supplementary file 1 [file ijms-23-06428-s001.zip › Supplementary Files/Supplementary Figures/Figure S4.jpg]

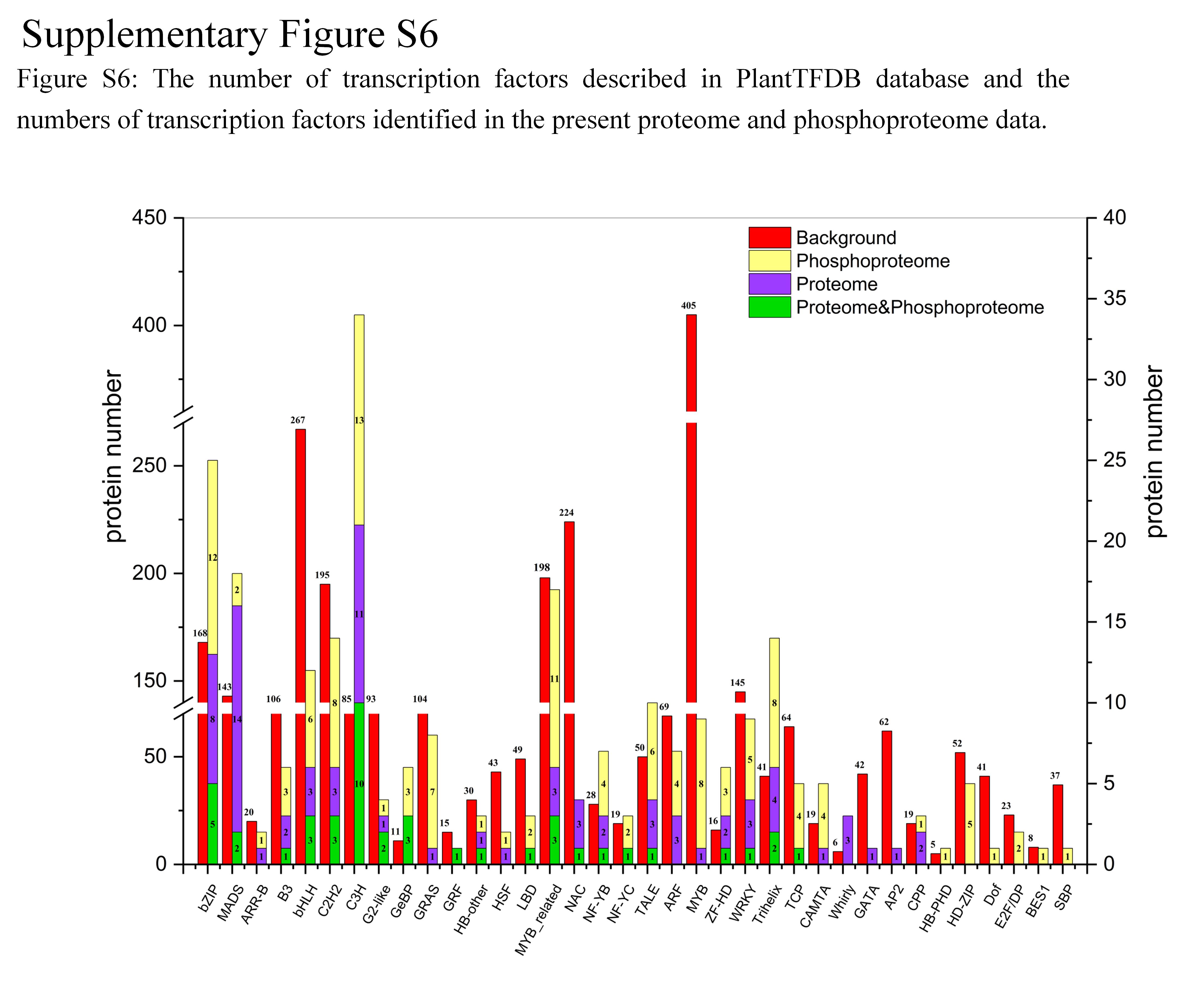

Supplement: Supplementary file 1 [file ijms-23-06428-s001.zip › Supplementary Files/Supplementary Figures/figure S6.jpg]
